# Pharmacological treatments of fibromyalgia in adults; overview of phase IV clinical trials

**DOI:** 10.3389/fphar.2022.1017129

**Published:** 2022-09-23

**Authors:** Nasser M. Alorfi

**Affiliations:** Department of Pharmacology and Toxicology, College of Pharmacy, Umm Al-Qura University, Makkah, Saudi Arabia

**Keywords:** fibromyalgia, pain, clinical trials, neuroscience, phase IV

## Abstract

**Background:** Fibromyalgia is a chronic neurological condition characterized by widespread pain. The effectiveness of current pharmacological treatments is limited. However, several medications have been approved for phase IV trials in order to evaluate them.

**Aim:** To identify and provide details of drugs that have been tested in completed phase IV clinical trials for fibromyalgia management in adults, including the primary endpoints and treatment outcomes. This article was submitted to Neuropharmacology, a section of the journal Frontiers in Pharmacology.

**Method:** Publicly available and relevant phase IV trials registered at ClinicalTrials.gov were analyzed. The uses of the trialed drugs for fibromyalgia were reviewed.

**Results:** As of 8 August 2022, a total of 1,263 phase IV clinical trials were identified, of which 121 were related to fibromyalgia. From these, 10 clinical trials met the inclusion criteria for the current study. The drugs used in phase IV trials are milnacipran, duloxetine, pregabalin, a combination of tramadol and acetaminophen, and armodafinil. The effectiveness of the current pharmacological treatments is apparently limited.

**Conclusion:** Due to its complexity and association with other functional pain syndromes, treatment options for fibromyalgia only are limited and they are designed to alleviate the symptoms rather than to alter the pathological pathway of the condition itself. Pain management specialists have numerous pharmacologic options available for the management of fibromyalgia.

## Introduction

Fibromyalgia (FM) is a complex condition affecting a person’s sensory processing system and it has a significant morbidity rate (Clauw, 2009; Sumpton and Moulin, 2014). Like other functional pain syndromes, fibromyalgia shows symptoms without pathologic findings. It is characterized by widespread musculoskeletal pain accompanied by anxiety, fatigue, cognitive dysfunction, and sleep disruption (Neumann and Buskila, 2003; Arnold et al., 2008; Häuser et al., 2015; Andrade et al., 2020). Other symptoms associated with fibromyalgia include tension headaches, temporomandibular joint (TMJ) disorders, irritable bowel syndrome, and depression; these ultimately lead to a decline in patients’ quality of life (Silver and Wallace, 2002; Ayouni et al., 2019; Santos et al., 2019). Some patients report morning stiffness and gastrointestinal irritation. Several asymptomatic conditions can also develop in fibromyalgia patients, such as osteoarthritis, hyperparathyroidism, degenerative disk disease, and calcifications (Maria De Freitas Trindade Costa et al., 2016; Maugars et al., 2021). The condition is reported in more women than in men (Yunus, 2001).

Clinically, the presence of fibromyalgia has been linked to an increased response to almost any type of stimulus, including hot, cold, electrical stimulation, photosensitivity, and sometimes the brightness of light or the volume of auditory tones. These all contribute to enhanced pain sensitivity and they induce persistent pain (Geisser et al., 2008; Becker and Schweinhardt, 2012; Martenson et al., 2016). The widespread pain experienced by individuals with fibromyalgia has been attributed to primarily central mechanisms such as central sensitization at the spinal level and abnormal pain processing in the brain (Desmeules et al., 2003; Cagnie et al., 2014). Although the exact cause of fibromyalgia is not fully understood, biological factors, mechanical/physical trauma or injury, genetic factors, and psychosocial stressors have been held responsible for the painful condition (Sommer et al., 2008; Amsterdam and Buskila, 2021). The disease is well known for the abnormal pain sensitivity that it causes, along with abnormal neurotransmitter levels in cerebrospinal fluid (CSF), abnormal activation of cerebral pain-processing areas, and abnormal peripheral pain and sensory thresholds (Larson et al., 2000; Staud and Domingo, 2001; Staud, 2002). These could be treatment targets for some drugs.

Despite its pathophysiological complexity, fibromyalgia may present with several comorbidities that worsen the sensation. Interestingly, the factors contributing to the pathophysiology of fibromyalgia may also exist with other functional pain syndromes such as functional abdominal pain syndrome, irritable bowel syndrome, chronic pelvic pain syndrome, and TMJ disorder (Shaver, 2008; Yunus, 2012; Johnson and Makai, 2018). This co-existence leads to further complexity for clinicians.

### Pharmacological targets in fibromyalgia treatment

Research advances are increasingly focusing on the development of analgesics and pain management medications that can be used for fibromyalgia. In this context, selective serotonin reuptake inhibitors, norepinephrine inhibitors, skeletal muscle relaxants, anti-epileptic agents, and anesthetics have been mainly investigated (Sarzi-Puttini et al., 2008; Häuser et al., 2009). Other targets for pain alleviation include the inhibition of excitatory neurotransmitters, substance P, and glutamate (Sarchielli et al., 2007; Owen, 2008; Guymer and Littlejohn, 2021). The aim of treatment for fibromyalgia is often to reduce pain-related symptoms. However, there is no specific pathophysiological therapeutic target.

### Pharmacological treatments

Fibromyalgia is a chronic, life-long condition, and it has no known etiology and no single cure. The multidisciplinary approach with medications, physiotherapy, psychologic, and other modalities provides the best chance of improved outcomes without promoting polypharmacy (Menzies et al., 2017; Oliveira Júnior and Ramos, 2019). Caution is advised when designing a treatment plan in fibromyalgia.

A considerable amount of literature has listed different pharmacological treatments used as adjunct medications, such as selective serotonin reuptake inhibitors, e.g., fluoxetine (Lawson, 2002; Häuser et al., 2015; Walitt et al., 2015); skeletal muscle relaxants, e.g., cyclobenzaprine (Tofferi et al., 2004); tramadol (Russell et al., 2000; Goldenberg et al., 2004); clonazepam (Corrigan et al., 2012); lidocaine (Abeles et al., 2007). Moreover, caffeine is used as a non-selective antagonism of adenosine receptors which reduce pain processing (Scott et al., 2017). Most of the above-mentioned drugs are used to induce sleep, reduce the symptoms of depression and anxiety and/or minimize fatigue. However, drugs that are US FDA approved specifically for fibromyalgia include milnacipran, duloxetine, and pregabalin.

This review focuses on medications that have been tested in phase IV clinical trials only. It identifies and summarizes the drugs that have been trialed for fibromyalgia and it provides an overview of phase IV clinical trials that assess a pharmacological treatment of fibromyalgia in adults. The drugs found to be used in phase IV trials on fibromyalgia are milnacipran, duloxetine, pregabalin, the combination of tramadol and acetaminophen, and armodafinil.

### Non-pharmacological treatments

Several studies have revealed that not only pharmacological treatments are available for fibromyalgia, but that non-pharmacological options could also help patients with this condition. For patients with chronic pain, a number of non-pharmacological options, including physical and aerobic exercises and cognitive-behavioral therapy (CBT), have shown promising results as standalone, adjunctive treatments (Hassett and Williams, 2011; Nüesch et al., 2013). A study conducted by Bernik et al. (2013) concluded that pharmacotherapy and CBT therapy should preferably be provided to all patients with fibromyalgia.

Moreover, studies suggest that psychological support is essential for patients with fibromyalgia due to the many negative emotions that can accompany the condition. Psychological treatments include mindfulness meditation, stress management and coping mechanisms, e.g., sleep hygiene and relaxation techniques improved pain perception and minimized pain symptoms (Hassett and Gevirtz, 2009; Aman et al., 2018). Indeed, complementary treatments including frequent movement, electroacupuncture, acupuncture, and chiropractor therapy are effective as adjuvant therapies to all patients with fibromyalgia (Sarac and Gur, 2005; Zheng and Faber, 2005; Martin et al., 2006; Ablin et al., 2013).

## Study design

ClinicalTrials.gov was searched to identify trials relevant to this review. ClinicalTrials.gov is an online database, effectively a public registry of clinical trials conducted in 221 countries; it contains information about medical studies with human volunteers. This review evaluated drugs used in fibromyalgia and it examined entries related to pharmacological fibromyalgia studies. See the following section for the parameters used in the search. Articles were screened independently by two reviewers and assessed for risk of bias. Data from 1,263 clinical trials were downloaded from ClinicalTrials.gov on 08/08/2022. After excluding trials that involved the treatment of other complications or were not at phase IV, 10 clinical trials remained as eligible for the review. The inclusion criteria were: 1) primary focus on fibromyalgia; 2) completed studies; 3) for patients of adult age (18–64 years old), and 4) results were available.

### Data Extraction

The data were extracted manually and downloaded from ClinicalTrials.gov, covering the following:

• Interventions: Details of interventional (clinical trial).
• Conditions: The medical condition treated was selected as “Fibromyalgia.”
• Trial design: Phase IV only.

The trials’ results were extracted manually from the results reported in the registry. The primary outcomes, number of participants, timeframe and results were collected.

## Results

The drugs used in the phase IV trials are milnacipran, duloxetine, pregabalin, a combination of tramadol and acetaminophen, and armodafinil. Interestingly, five out of the ten clinical trials were used to evaluate the role of milnacipran in fibromyalgia. The official title of the trial, the intervention, aims, primary measures (endpoints) and timeframe are shown in Table 1.

**TABLE 1:** (Data from https://clinicaltrials.gov, updated on 08-08-2022).

No.	Study title	Treatment	Aim	*n*	Primary measures (endpoints)	Time frame
1	Effects of a 12-Week milnacipran 200 mg treatment on pain perception and pain processing in fibromyalgia—An open-label study	Milnacipran	To investigate the effects of milnacipran treatment on neurotransmitter release in fibromyalgia	8	The concentration of Substance P in cerebrospinal fluid in response to experimental pain before and after Milnacipran treatment	12 weeks
2	A randomized, double-blind, placebo-controlled, two-way crossover study to evaluate the effect of milnacipran on pain processing and functional magnetic resonance imaging activation patterns in patients with fibromyalgia	Milnacipran	To evaluate the effects of milnacipran on pain processing and functional mri in patients with fibromyalgia	22	1) Concentration of Substance P in cerebrospinal fluid in response to experimental pain before and after milnacipran treatment	16 weeks
					2) Measure the sensory threshold for temperature pain and pressure pain	
					3) Measure pain ratings and fibromyalgia symptoms	
					5) Measure concentrations of serotonin and norepinephrine cerebrospinal fluid and plasma	
3	A multicenter, randomized, double-blind, placebo-controlled switch study to evaluate the safety, tolerability and efficacy of milnacipran in patients with an inadequate response to duloxetine for the treatment of fibromyalgia	Milnacipran	To evaluate the safety, tolerability and efficacy of milnacipran in patients with an inadequate response to duloxetine for the treatment of fibromyalgia	107	1) Responder status based on patient global impression of change (PGIC) Score at Visit 5 (Week 13)	12 weeks
					2) Change from baseline to visit 5 (Week 13) in the visual analog scale (VAS) 1-week pain recall score	
4	The effects of milnacipran on sleep disturbance in fibromyalgia	Milnacipran	The study aimed at examining the effects of milnacipran on sleep disturbance in patients with fibromyalgia	19	The primary endpoints were the difference in sleep maintenance defined by PSG-recorded wake after sleep onset (WASO), number of awakenings after sleep onset (NAASO), and SE	4 weeks
5	A multicenter, randomized, double-blind, placebo-controlled discontinuation study of the durability of effect of milnacipran for the treatment of fibromyalgia in patients receiving long-term milnacipran treatment	Milnacipran	To evaluate the durability of effect of milnacipran for the treatment of fibromyalgia in patients receiving long-term milnacipran treatment and to characterize the effects of milnacipran on multiple symptoms of fibromyalgia, as demonstrated by changes in symptoms following the discontinuation of milnacipran	340	1) Time to loss of therapeutic response (LTR)	17 weeks
					2) Time to worsening in patient global impression of change (PGIC)	
					3) Time to worsening in multidimensional assessment of fatigue (MAF)	
6	Flexible dosed duloxetine versus placebo in the treatment of fibromyalgia	Duloxetine hydrochloride	To confirm the efficacy and safety of duloxetine 60–120 mg once daily in comparison to placebo on symptom improvement in patients meeting criteria for fibromyalgia aged 18 and older	530	1) Patient’s global impressions of improvement (PGI-I) at Week 12	12 weeks
					2) Change from baseline in brief pain inventory (BPI) (Modified Short Form), Multidimensional fatigue inventory (MFI), Clinical global impressions of severity (CGI-S), Beck anxiety inventory (BAI), 36-Item Short-form Health Survey (SF-36), Massachusetts General Hospital Cognitive and Physical Functioning Questionnaire (MGH-CPFQ), the Mood, Anxiety, Pain, Sleep, and Stiffness Likert Scale, blood pressure and heart rate at 12 Week Endpoint	
					3) Number of responders: 30% and 50% improvement in brief pain inventory average pain at 12 week endpoint	
7	A randomized, double-blind comparison of duloxetine 30 mg QD and placebo in adult patients with fibromyalgia	Duloxetine	To determine if 30 milligrams (mg) of duloxetine is effective in the treatment of fibromyalgia compared to placebo	308	1) Change in weekly average pain intensity	12 weeks
					2) Change in evoked pain scores	
					3) Identification of group assignment	
8	A 6-month, open-label, safety trial of pregabalin in adolescent patients with fibromyalgia	Pregabalin	To evaluate the long-term safety of pregabalin in adolescent patients who participated in the previous fibromyalgia study (A0081180) and who wish to receive open-label pregabalin	63	Change from baseline in pain numeric rating scale by week	24 weeks
9	Ultracet [Tramadol HCL (37.5 mg)/acetaminophen (325 mg)] combination tablets in the treatment of the pain of fibromyalgia	Tramadol and acetaminophen	To evaluate the analgesic effect of combination of tramadol hydrochloride and acetaminophen in participants for treatment of fibromyalgia pain (chronic widespread pain and presence of tender points)	80	Pain visual analog scale score at day 14, 28 and 56	8 weeks
10	An 8 week, double-blind efficacy study of armodafinil augmentation to alleviate fibromyalgia fatigue	Armodafinil	To determine if armodafinil is safe and tolerable in the treatment of FM-induced fatigue	55	Brief Fatigue Inventory	8 weeks

### Milnacipran

Milnacipran is a non-tricyclic compound that inhibits the re-uptake of both serotonin and norepinephrine (Yoshida et al., 2004; Häuser et al., 2015). As a result, serotonin and norepinephrine levels are increased and disorders resulting from a lack of these neurotransmitters are improved. This leads to a reduction in symptoms, including pain, fatigue, and cognitive deficits. A three-fold greater selectivity for norepinephrine inhibition is found with milnacipran than for serotonin inhibition (Palmer et al., 2010; Raouf et al., 2017). The drug is approved by the US Food and Drug Authority (FDA) exclusively for fibromyalgia. Milnacipran is believed to inhibit some excitatory neurotransmitters such as substance P, which may result in the reduction of pain severity. It is recommended for the treatment of various chronic pain syndromes (Elliott et al., 2009; Kyle et al., 2010). It can also be effective for fibromyalgia patients with coexisting depression (Ormseth et al., 2010). A drug evaluation paper concluded that milnacipran gives modest pain relief in fibromyalgia patients and is best used as part of a multidisciplinary treatment strategy (Bernstein et al., 2013). The results of another study showed that milnacipran significantly reduced pain scores, helped patients to achieve lower mean global impression scores, and significantly increased response rates, regardless of the depressive symptoms at baseline (Arnold et al., 2012). Milnacipran has some major but rare side effects such as worsening suicide risk and causing liver damage (Montgomery and Briley, 2010; Park and Ishino, 2013). Moreover, some common side effects include gastrointestinal upset and urinary disorders (Tignol et al., 1998; Levin, 2016). Interestingly, milnacipran does not inhibit the cytochrome P 450 system, which reduces the chances of possible interactions with other drugs (Pae et al., 2009; Paris et al., 2009). The five studies that have used milnacipran in phase IV trials to treat fibromyalgia are shown in Table 1.

### Duloxetine

Duloxetine is a serotonin–norepinephrine reuptake inhibitor (SNRI) (Westanmo et al., 2005; JS et al., 2019). Pre-clinical studies show that duloxetine can alleviate diabetic peripheral neuropathic pain, fatigue, pain and other related symptoms (Wernicke et al., 2006). It has also been found to significantly reduce pain and improve functioning in patients with chronic low back pain (Skljarevski et al., 2010). In patients with fibromyalgia, duloxetine improved average pain severity and self-reported improvement (Chappell et al., 2008). It has also been found to be effective in the long-term treatment of fibromyalgia (Mease et al., 2010). The study by Mease et al. (2010) reported that dry mouth and nausea were the most reported side effect, but duloxetine is generally safe and well tolerated, including in older patients and those with concomitant illnesses (Wernicke et al., 2005). It is preferably avoided in patients with hepatic insufficiency.

### Pregabalin

Pregabalin, the first of the three medications that have gained US FDA approval for fibromyalgia, is an α2 *δ* ligand that acts by binding the α2 *δ* subunit of voltage-gated calcium channels (Micó and Prieto, 2012). This leads to a decrease in calcium influx at nerve terminals, with a consequential modulation in excitatory neurotransmitter release pain-related neurotransmitters, including glutamate and substance P (Tanabe et al., 2008; Alles et al., 2020). Studies have also suggested that pain associated with major depressive disorder can be reduced with pregabalin (Showraki, 2007; Stein et al., 2008; Frampton, 2014). Previous studies concluded that pregabalin was generally well tolerated in the long-term treatment of anxiety disorders (Feltner et al., 2008; Montgomery et al., 2013). Generally, pregabalin is safe and well tolerated (Durkin et al., 2010). However, number of uncomfortable side effects have been reported with pregabalin, although these tend to be transient and dose dependent (Zin et al., 2010; Toth, 2014). Only a single clinical trial was available at phase IV as shown in Table 1.

### Tramadol and acetaminophen

Tramadol is a centrally acting, fully synthetic opioid and one of the most commonly used central nervous system analgesics (Leppert, 2009; Duehmke et al., 2017). It is an effective and well tolerated agent that is taken to reduce pain (Minami et al., 2015; Nakhaee et al., 2021). Tramadol can be used as a second-line treatment for more resistant cases in fibromyalgia patients and it has a positive effect on fibromyalgia pain (Maclean and Schwartz, 2015; Pereira Da Rocha et al., 2020). On the other hand, acetaminophen is a central analgesic drug that is mediated through the activation of descending serotonergic pathways (Pickering et al., 2008; Jozwiak-Bebenista and Nowak, 2014). Only one single clinical trial was available at phase IV as shown in Table 1. In fibromyalgia, a combination of tramadol and acetaminophen was effective for the treatment of fibromyalgia pain without any serious adverse effects (Bennett et al., 2003).

### Armodafinil

Armodafinil is the R-enantiomer of racemic modafinil, a wakefulness-promoting medication, that primarily affects the areas of the brain involved in controlling wakefulness (Hirshkowitz et al., 2007; Garnock-Jones et al., 2009). The mechanism of action of armodafinil is not completely understood. It is mainly used for the treatment of excessive sleepiness associated with narcolepsy, and obstructive sleep apnea (Lankford, 2008; Schwartz et al., 2010). The potential role of stimulants such as armodafinil is often to manage the fatigue that is a common symptom of fibromyalgia. It has been used to improve menopause-related fatigue (Meyer et al., 2016) and sarcoidosis-associated fatigue (Lower et al., 2013). However, only a single clinical trial was available at phase IV for fibromyalgia management. The study concluded that there was no significant difference in any effectiveness outcome (Thomas et al., 2010).

## Discussion

Fibromyalgia is a chronic, life-long condition with no single cure. There is no one treatment that can treat all the symptoms of fibromyalgia since the condition has many symptoms. Pain severity, global severity and physical functioning are significantly and negatively influenced by fibromyalgia. Also, psychological factors such as anxiety, stress, and depression exacerbate the condition. The treatments being assessed in the 10 trials reviewed here aimed to improve several health parameters, including physical fitness, work and other functional activities, and mental health. This review focused on medication that was tested in phase IV clinical trials only. The drugs used in phase IV trials for fibromyalgia are milnacipran, duloxetine, pregabalin, a combination of tramadol and acetaminophen, and armodafinil. The US FDA has approved three drugs to treat fibromyalgia: milnacipran, duloxetine and pregabalin, as shown in Table 2. Of these milnacipran is exclusively for the management of fibromyalgia. Both milnacipran and duloxetine act as selective serotonin and norepinephrine re-uptake inhibitors. A considerable amount of research concluded that patients with fibromyalgia have abnormally low levels of serotonin, noradrenaline and dopamine in their brains, which leads to serious side effects including the worsening of fibromyalgia status. Serotonin regulates numerous bodily functions. In adult patients with fibromyalgia, milnacipran, duloxetine and pregabalin were associated with significant improvements in pain and other symptoms. One of the considerations that should be included in future investigations is the combined therapy of pharmacological and non-pharmacological treatments to manage fibromyalgia symptoms. Further clinical trials investigating the efficacy and safety of treatments used for fibromyalgia are warranted.

**TABLE 2:** FDA-approved drugs for the treatment of fibromyalgia.

Treatment	Mechanism of action	Medication class	Indication	Side effect
Milnacipran	A selective norepinephrine reuptake inhibitor with a three times greater selectivity for norepinephrine reuptake inhibition over serotonin reuptake inhibition	Selective serotonin and norepinephrine reuptake inhibitors (SSNRIs)	Fibromyalgia	GI Upset
				Palpitations, increased heart rate
				Dry mouth
				Increases blood pressure
Duloxetine	Inhibits the reuptake of serotonin and norepinephrine (NE) in the central nervous system	Selective serotonin and norepinephrine reuptake inhibitors (SSNRIs)	Major depressive disorder	Increases blood pressure
			Generalized anxiety disorder	
			Diabetic peripheral neuropathic pain	Hepatitis
			Fibromyalgia	Cholestatic
			Chronic musculoskeletal pain in adults	
Pregabalin	Potentiates GABA activity	Anticonvulsants	Neuropathic pain	Sedation
			Postherpetic neuralgia	
			Partial-onset seizures	
			Fibromyalgia	

## Conclusion

The findings from the review suggest that the drugs most commonly used for fibromyalgia that can be considered first-line options are milnacipran, duloxetine, and pregabalin. The number of trials for fibromyalgia are extremely limited Pharmacological options can be employed to provide patients with a better quality of life. Despite years of investigation of pharmacological treatments for fibromyalgia, non-pharmacological treatments show promise for future investigation.
